# Evaluating the impact of immunization with the “pan-fungal” vaccine, NXT-2, on the gut mycobiome and microbiome in non-human primates (NHPs)

**DOI:** 10.1128/spectrum.01047-26

**Published:** 2026-07-15

**Authors:** Kwadwo O. Oworae, Whitney Rabacal, Anna Hu, Daniel A. Wychrij, Emily Rayens, Taylor I. Chapman, Justin Bahl, Karen A. Norris

**Affiliations:** 1 Center for Vaccines and Immunology, University of Georgia 1355, Athens, Georgia, USA; 2 Department of Infectious Diseases, University of Georgia 1355, Athens, Georgia, USA; 3 Department of Research & Evaluation, Kaiser Permanente Southern California 82579, Pasadena, California, USA; USDA-ARS Arkansas Children's Nutrition Center, Little Rock, Arkansas, USA

**Keywords:** *Candida*, *Aspergillus*, pan-fungal vaccine, NXT-2, microbiome, mycobiome, non-human primates

## Abstract

Fungal infections remain a significant public health concern with high mortality, morbidity, and increasing associated health costs. This burden is projected to rise due to expansion of at-risk populations, limited therapeutics, increasing drug resistance, and the emergence of new fungal pathogens. Even with these challenges, there are currently no approved vaccines. We previously developed a “pan-fungal” vaccine candidate, NXT-2, that confers protection against multiple invasive fungal infections such as pulmonary aspergillosis, pneumocystosis, and invasive candidiasis, as well as non-invasive vulvovaginal candidiasis. NXT-2 is a 90 amino acid consensus peptide designed from a conserved region of the fungal antigen (KEX1). We assessed the effect of NXT-2 immunization on gut microbial diversity, composition, and functional capacity in non-human primates. To do this, we monitored changes in the gut mycobiome and microbiome pre- and post-vaccination using ITS2 and metagenomic sequencing, respectively, in Japanese and rhesus macaque cohorts. NXT-2 elicited a robust antibody response without disrupting the gut microbial communities in both macaque species. The mycobiome exhibited stability with no significant changes in alpha and beta diversity, taxonomic composition, or functional guild distributions. The relative abundance of gut resident *Candida* and *Aspergillus* species remained stable and was not significantly altered following vaccination. The microbiome showed stability with preserved alpha and beta diversities, taxonomic composition, and functional capacity. Results from this study show the first cross-kingdom analysis demonstrating that antifungal vaccination can achieve protective immunity without perturbing gut microbial communities. This establishes a framework for microbiome-informed vaccine assessment beyond conventional immunogenicity and adverse effect monitoring.

**IMPORTANCE:**

Fungal infections cause millions of deaths annually, yet no vaccines are approved despite growing drug resistance and limited treatment options. NXT-2 is a pan-fungal vaccine that protects against multiple fungal infections such as pneumocystosis, candidiasis, and aspergillosis. Here, we demonstrate in NHPs that NXT-2 elicits robust protective antibody responses without altering gut bacterial or fungal communities. This is the first study to assess antifungal vaccination across both microbial kingdoms and establish that protective antifungal immunity can be achieved while preserving resident microbiota. This work provides a framework for incorporating microbiome assessment into vaccine development beyond conventional immunogenicity and adverse event monitoring.

## INTRODUCTION

Fungal infections (FIs) remain a serious public health concern with increasing morbidity and mortality (1–3). Invasive fungal infections (IFIs), which are life-threatening account for about 6.5 million cases yearly, resulting in over 3.7 million deaths while non-invasive fungal infections (NIFIs) account for the higher morbidity with over 1 billion affected individuals (1, 4). These numbers are projected to rise due to the increase in at-risk populations, the increase in incidence of drug-resistant pathogens, and the emergence of new fungal pathogens as a result of climate change (5, 6). In the USA, over $11.5 billion is lost to direct medical costs of fungal infections, including diagnosis and treatment and indirect cost such as loss in labor (7, 8).

Despite the significant clinical burden of FIs, there are no clinically approved fungal vaccines. To address the growing public health concern, our laboratory developed a “pan-fungal” vaccine candidate, NXT-2. NXT-2 is a recombinant protein designed as a consensus protein based on the conserved amino acid sequence of the KEX1 protein of the major pathogenic fungal organisms (9). Development of a “pan-fungal” vaccine is a challenging but clinically important strategy as individuals at risk of FIs are susceptible to infections by multiple fungal pathogens (7, 10, 11). We have demonstrated the immunogenicity and protective efficacy of NXT-2 immunization against multiple experimental IFIs, including systemic candidiasis (due to *Candida albicans* and *Candidozyma auris*), pulmonary aspergillosis, and *Pneumocystis* in immunosuppressed mice and non-human primates (NHPs) (9, 12, 13), as well as NIFIs such as vulvovaginal candidiasis in a murine model (14). We have demonstrated that antibody-mediated protection is induced by NXT-2 vaccination in both systemic and localized fungal infections (9, 13–15).

It is important to not only evaluate NXT-2 in the context of immunogenicity and pathogen clearance but also understand the potential impact of vaccination on the host commensal microbial ecosystems (16). The human gut harbors a diverse and dynamic community of microorganisms, including bacteria, viruses, archaea, and fungi (17). While bacteria are numerically dominant (comprising over 90% of the gut microbiota) and have been the main focus of microbiome studies, the mycobiome (fungal component) is an important but understudied contributor to host immune regulation and health (18, 19). Fungi represent less than 0.1% of the gut microbiota, but they engage in essential interactions with both the host and co-resident bacteria in maintaining mucosal immunity and modulating inflammation (19–21). The gut mycobiome and microbiome contribute to immune homeostasis, pathogen resistance, and mucosal barrier function (17, 19–22). Disruptions in these communities have been associated with various adverse outcomes, including inflammatory bowel disease, metabolic dysfunction, autoimmune diseases, and increased susceptibility to infections (19, 20, 22).

Despite the importance of the gut microbial ecosystem on the host immune homeostasis, studies on the impact of immunization on the gut microbiome remain limited. Studies on viral vaccines have largely shown minimal or transient effects on gut microbial composition (23–25). However, a growing body of evidence suggests that the gut microbiome may play a more active role in shaping the host’s response to vaccination by modulating immunogenicity, durability, and overall vaccine efficacy (26–28). Furthermore, the gut microbial composition is known to be remarkably stable and resilient, particularly in healthy individuals, with its composition tending to recover after perturbations such as dietary changes, short illness, or even antibiotic use (29, 30). This ecological robustness suggests that the risk of long-term microbiome and mycobiome disruption by vaccination may be low. Nevertheless, because NXT-2 vaccine is derived from a conserved fungal antigen (KEX1), we addressed whether vaccine-induced immunity altered the abundance or diversity of beneficial gut fungi or disrupted interkingdom interactions between fungi and bacteria. In this study, we assessed the impact of NXT-2 vaccination on the gut microbiome and mycobiome in NHPs using ITS2 and metagenomic sequencing, respectively. NHPs share close similarities with humans in genetics, physiology, and immune responses, making them a relevant model for biomedical research (31). The gut microbiome composition of NHPs has also been shown to closely resemble that of humans (32, 33), and they have been widely used to improve our understanding of gut microbial ecosystems and host-microbiome interactions. This study provides critical insights into the ecological stability of the gut microbiome and mycobiome following of NXT-2 immunization and offers a comprehensive framework for evaluating fungal vaccines in the future.

## MATERIALS AND METHODS

### Animals

Japanese macaques (*Macaca fuscata*) aged 6–9 years (*n* = 7; 1 male, 6 females) were obtained from Oregon National Primate Research Center (Hillsboro, OR). Rhesus macaques (*Macaca mulatta*) aged 3 years (*n* = 5; 3 males, 2 females) were obtained from Southwest National Primate Research Center (San Antonio, TX). All animals were housed at the University of Georgia primate facilities in accordance with the NIH Guide for the Care and Use of Laboratory Animals. The study protocol was reviewed and approved by the Institutional Animal Care and Use Committee (IACUC) at the University of Georgia. The University of Georgia is accredited by the Association for Assessment and Accreditation of Laboratory Animal Care (AAALAC). All animals were housed in a 12-h light-dark schedule and given *ad libitum* access to primate chow (Lab Diet 5038, St. Louis, MO) with supplementation of fresh fruits and vegetables.

### Immunization and sample collection

We utilized an affinity tag-free variant of NXT-2, called NXT-2a. The NXT-2a vaccine construct design, purification, and vaccination were performed as described (13). All animals were vaccinated intramuscularly with a dose of 100 µg of recombinant NXT-2a antigen formulated with Allhydrogel adjuvant 2% (Invivogen, San Diego, CA). A second dose was administered 6 weeks (Japanese macaque cohort) or 4 weeks (rhesus macaque cohort) after the primary vaccination. Fecal samples were collected 1 week prior to the primary vaccination (pre-vaccination) and 2 weeks following the booster immunization (post-vaccination). We chose 1 week before vaccination as the baseline and 2 weeks after immunization to assess the vaccine’s effect at peak antibody titers. Blood samples were taken at pre-vaccination and post-vaccination at 2-week intervals. Blood was centrifuged at 3,000 × *g* for 5 min at room temperature, and plasma samples were stored at −80°C for downstream analysis. Body weight was recorded throughout the study. No adverse effects, including changes in body weight, stool patterns, or gastrointestinal symptoms, were observed in any animal following vaccination.

### NXT-2 ELISA

Plasma NXT-2 antibody titers were evaluated using ELISA assay, as previously described (13). Briefly, microtiter plates (ThermoFisher) were coated with 5 µg/mL of purified NXT-2a and incubated overnight at 4°C. To determine the reciprocal endpoint titer (RET), heat-inactivated (56°C, 30 min) plasma samples were initially diluted at 1:1,000 and then serially diluted twofold. The plates were incubated overnight at 4°C. Plates were washed four times with PBS-T and incubated with goat anti-monkey IgG-HRP secondary antibody (1:10,000; Nordic Immunology) for 1 h at 37°C. After six washes with PBS-T, plates were developed with TMB substrate (BD Biosciences), stopped with 1 M H₂SO₄, and read at 450 nm. Data are reported as NXT-2 plasma IgG reciprocal endpoint titers (RET). Normal macaque plasma served as a negative control, and archival vaccinated samples with known titers served as positive controls.

### Metagenomic and ITS (Internal transcribed spacer) sequencing

Fecal samples were processed and sequenced by using Shotgun Metagenomic Sequencing Service and ITS Amplicon Sequencing Service (Zymo Research, Irvine, CA). For fungal mycobiome profiling, the internal transcribed spacer 2 (ITS2) region was amplified using the Microbiome Sequencing Services ITS2 Primer Set and sequenced on an Illumina NextSeq 2000 platform. Shotgun metagenomic sequencing was performed on NovaSeq platform for microbiome profiling (Illumina, San Diego, CA).

### ITS sequencing analysis

Raw paired-end ITS2 amplicon sequences were processed using QIIME2 (version 2024.10). Quality filtering and denoising were performed using DADA2 with forward and reverse reads truncated at 280 bp based on quality score profiles. Amplicon sequence variants (ASVs) were generated, and chimeric sequences were removed. Non-fungal sequences were filtered out by retaining only ASVs classified to the kingdom Fungi. **Taxonomic assignment** of fungal ASVs was performed using a pre-trained Naive Bayes classifier against the UNITE fungal ITS database (version 10.0, 99% clustered). ASVs were classified from the Kingdom through the Species level where possible. Samples with insufficient fungal read counts were excluded to maintain data quality. Both pre- and post-vaccination samples from the same animal were removed together to preserve the paired study design. **Alpha diversity** (Observed Features and Shannon diversity index) was computed to assess within-sample diversity. **Beta diversity** was assessed using Bray-Curtis dissimilarity and Jaccard distance matrices. Principal Coordinates Analysis (PCoA) was performed to visualize differences in community composition between samples. **Differential abundance** testing was conducted using ANCOM-BC2 (Analysis of Composition of Microbiomes with Bias Correction 2) to identify fungal taxa that differed significantly between pre- and post-vaccination timepoints. Taxa were filtered to include only those present in at least 20% of samples within at least one group and with at least 10 total reads across all samples. Analyses were performed at multiple taxonomic levels (Phylum through ASV). **Functional guild assignments** were performed using FUNGuild (Fungi Functional Guild database) to assign ecological roles to fungal taxa from ITS2 amplicon sequencing data. Genus-level taxonomic assignments were matched against the FUNGuild database to identify trophic modes (multitrophic, pathotroph, and saprotroph combinations). Guild assignments with high confidence rankings were retained for analysis to ensure ecological assignment accuracy. ASV-level abundances were aggregated by trophic mode category, normalized to relative abundances (%), and stratified by NHP subject and vaccination timepoint.

### Shotgun metagenomic sequencing analysis

Raw paired-end shotgun sequencing reads (2 × 150 bp) were quality-filtered using fastp (version 0.23.4) with a minimum Phred quality score Q20 and post-trimming length 50 bp. **Taxonomic classification** was performed using Kraken2 (version 2.1.3) against the standard Kraken2 database (version 20240906). Abundance estimation was refined using Bracken (version 2.9) with a read length parameter of 150 bp. Taxonomic abundance tables were imported into R and processed using phyloseq. Non-bacterial taxa were removed, including archaea, viruses, fungi, and eukaryotic sequences. **Diversity metrics** and **differential abundance analysis** were performed as described earlier. **Functional profiling** was performed using HUMAnN3 (v3.9) to characterize bacterial metabolic and functional potential from metagenomic sequencing data. Taxonomic prescreening was conducted using MetaPhlAn (v4.1.1) with the vJun23 database to identify species-level community composition. Nucleotide alignment was performed against the ChocoPhlAn pangenome database, followed by translated search of unmapped reads against the UniRef90 protein database using DIAMOND. Gene families were regrouped into KEGG Orthologs (KOs) and Gene Ontology (GO) terms, representing metabolic pathways and functional categories, respectively. KOs were further classified into four top-level KEGG categories (Metabolism, Genetic Information Processing, Environmental Information Processing, and Cellular Processes), while GO terms were organized into three domains (Biological Process, Molecular Function, and Cellular Component) (34, 35). Abundances were stratified by NHP subject and vaccination timepoints.

### Statistical analysis

All statistical analyses and data visualization were performed in GraphPad Prism (v10.6.1) and R (v4.4.1) using the phyloseq (v1.48.0), vegan (v2.6-8), ANCOMBC (v2.6.2), dplyr (v1.1.4), and ggplot2 (v3.5.1) packages. Statistical significance was set at *α* = 0.05 for all tests. Differences in alpha diversity between pre- and post-vaccination timepoints were assessed using paired Wilcoxon signed-rank tests to account for the repeated-measures design. PERMANOVA was used to test for significant differences in beta diversity. Taxa were considered differentially abundant if they had an FDR-adjusted *P* value < 0.05 in ANCOM-BC2 analysis.

## RESULTS

### NXT-2 immunization elicits a robust antibody response in both Japanese and rhesus macaques

**Fig 1:**
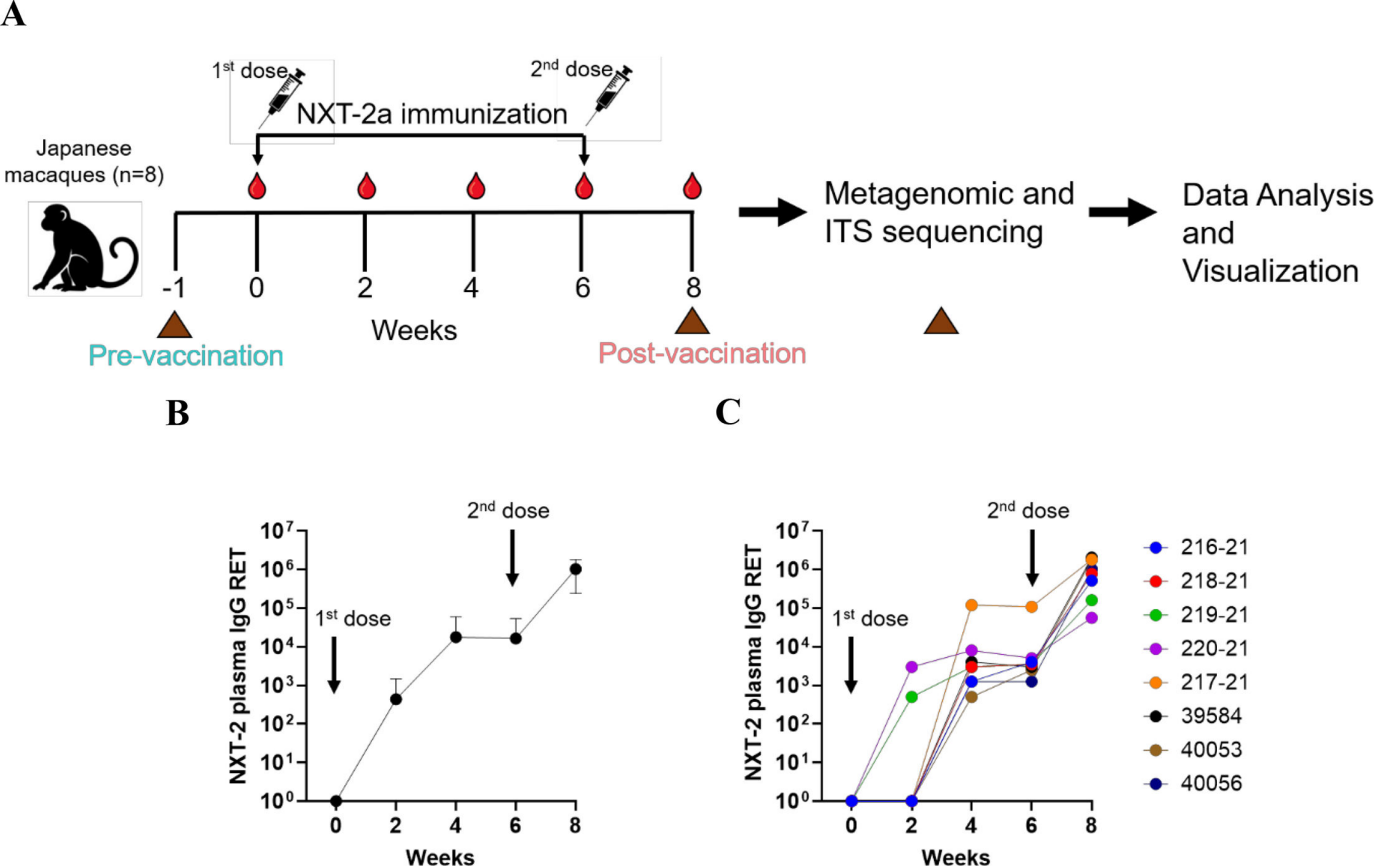
NXT-2 immunization elicits robust humoral responses in Japanese macaques. (**A**) Study design showing timeline for NXT-2 immunization (1st and 2nd dose), fecal sample (brown triangles) collection [Pre-vaccination (week −1) and post-vaccination (week 8)], blood collection (red drops), and downstream analysis. NXT-2-specific plasma IgG responses measured as reciprocal endpoint titers (RET) over time; (**B**) mean titer ± SD and (**C**) individual animal responses.

To assess the impact of NXT-2 vaccination on the gut mycobiome and microbiome, fecal samples were collected from eight Japanese macaques at pre-vaccination (week −1) and post-vaccination (week 8) timepoints following a two-dose vaccination regimen administered at weeks 0 and 6 (Fig. 1A). Blood samples were collected longitudinally to assess vaccine immunogenicity. The fecal samples were subjected to shotgun metagenomic and ITS sequencing methods for bacterial and fungal community analyses, respectively (Fig. 1A). A similar study was performed for the second cohort of rhesus macaques (Fig. S1A). In the Japanese macaques, NXT-2 plasma IgG RET increased from 1.76 × 10⁴ ± 4.14 × 10⁴ RET at 4 weeks after the first dose to 1.02 × 10⁶ ± 0.78 × 10⁴ RET following the boost in Japanese macaques (Fig. 1B). Individual animals showed consistent antibody responses across the cohort (Fig. 1C). Similar immunogenicity was observed in rhesus macaques, increasing from 1.23 × 10⁴ ± 0.81 × 10⁴ RET 4 weeks after the first dose to 2.88 × 10⁵ ± 1.02 × 10⁵ RET 2 weeks after the boost (Fig. S1B). Individual rhesus macaques exhibited comparable humoral responses to vaccination (Fig. S1C). These results confirm that NXT-2 vaccination elicits robust antibody responses in both macaque species.

### Mycobiome diversity remained stable following NXT-2 immunization

**Fig 2:**
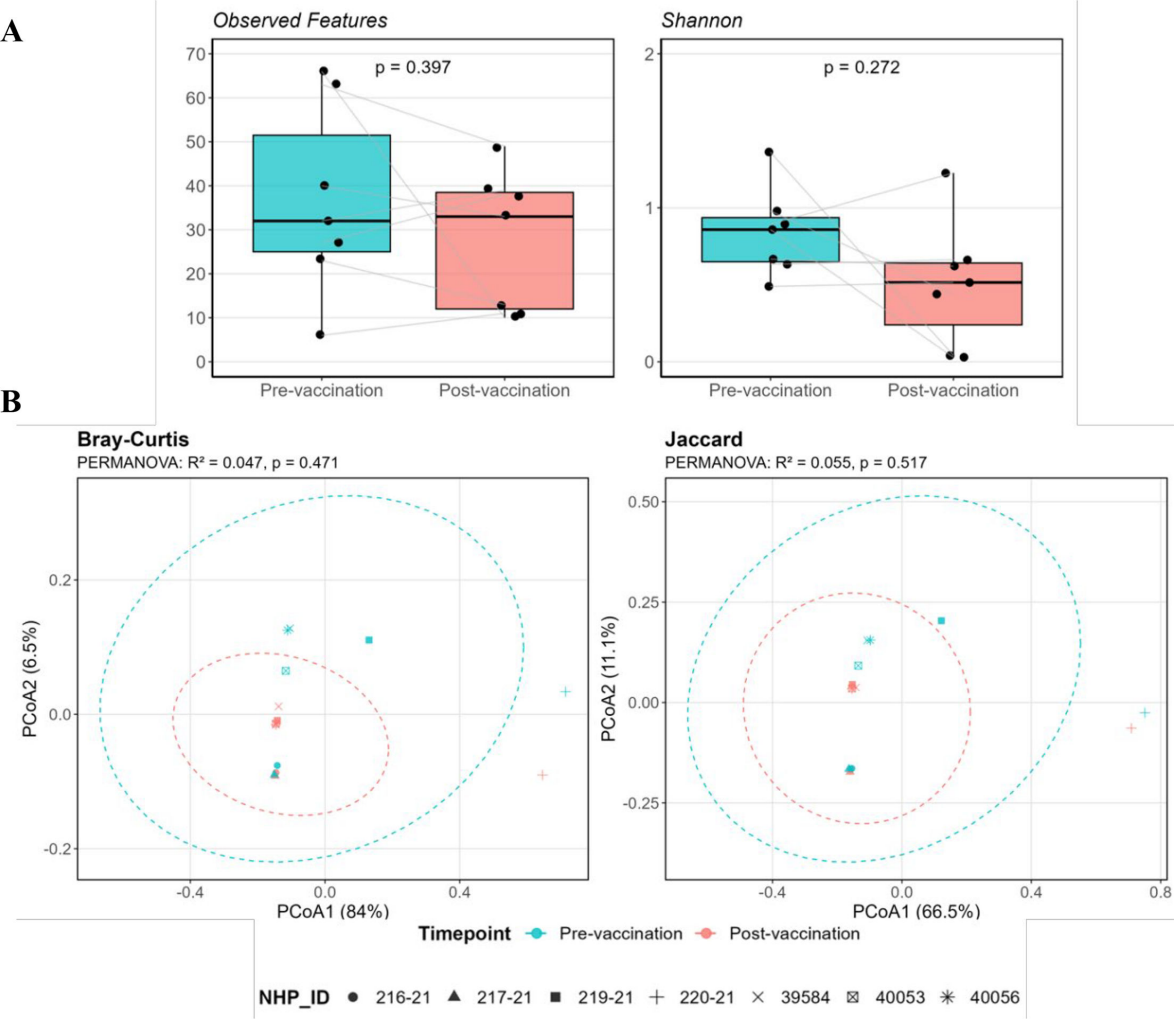
The gut fungal community structure remains stable following NXT-2 immunization. (**A**) Alpha diversity metrics (Observed Features and Shannon) showed no significant changes between pre- and post-vaccination timepoints in Japanese macaques (*n* = 7). Individual animals are shown as points connected by lines. Statistical comparison was performed using the Wilcoxon signed-rank test. (**B**) Beta diversity analysis (Bray–Curtis and Jaccard) visualized by PCoA showed no significant community shift. Individual samples are shown as shapes and colored by timepoint with 95% confidence ellipses. Group differences were assessed using PERMANOVA.

To assess whether NXT-2 vaccination altered the gut fungal community and composition, alpha and beta diversity analyses were performed. One sample from the Japanese macaque cohort was excluded after rarefaction, and the remaining samples were normalized to 17,000 sequences. For the alpha diversity metrics, the average observed features were 36 (range: 6–66) for pre-vaccination and 33 (range: 10–49) for the post-vaccination timepoint. This change was not statistically significant (Fig. 2A, *P* = 0.397). With the Shannon index, there was a decrease from 0.85 (range: 0.49–1.37) to 0.52 (range: 0.03–1.23) in the pre- and post-vaccination timepoints, respectively. This decrease was not significant (Fig. 2A, *P* = 0.272). Similarly, there was no significant difference in the observed features between vaccination timepoints in the rhesus macaques (Fig. S2A, pre-vaccination = 52 [range: 27–75], post-vaccination 39 [range: 30–77], *P* = 0.59). For the beta diversity metrics, both the Bray-Curtis dissimilarity and Jaccard indices showed no significant shifts in fungal community composition following NXT-2 vaccination (Fig. 2B, Bray-Curtis, *P* = 0.471; Jaccard, *P* = 0.517, respectively). PCoA plots also showed substantial overlap between timepoints in both plots (Fig. 2B). Analysis of the rhesus cohort supported these results, showing substantial overlap and no significant shifts in community composition by Bray-Curtis dissimilarity index (Fig. S2B, *P* = 0.058). Collectively, these results show the gut mycobiome diversity and structure remained stable after NXT-2 vaccination.

### Mycobiome taxonomic composition remains stable with no differentially abundant taxa

**Fig 3:**
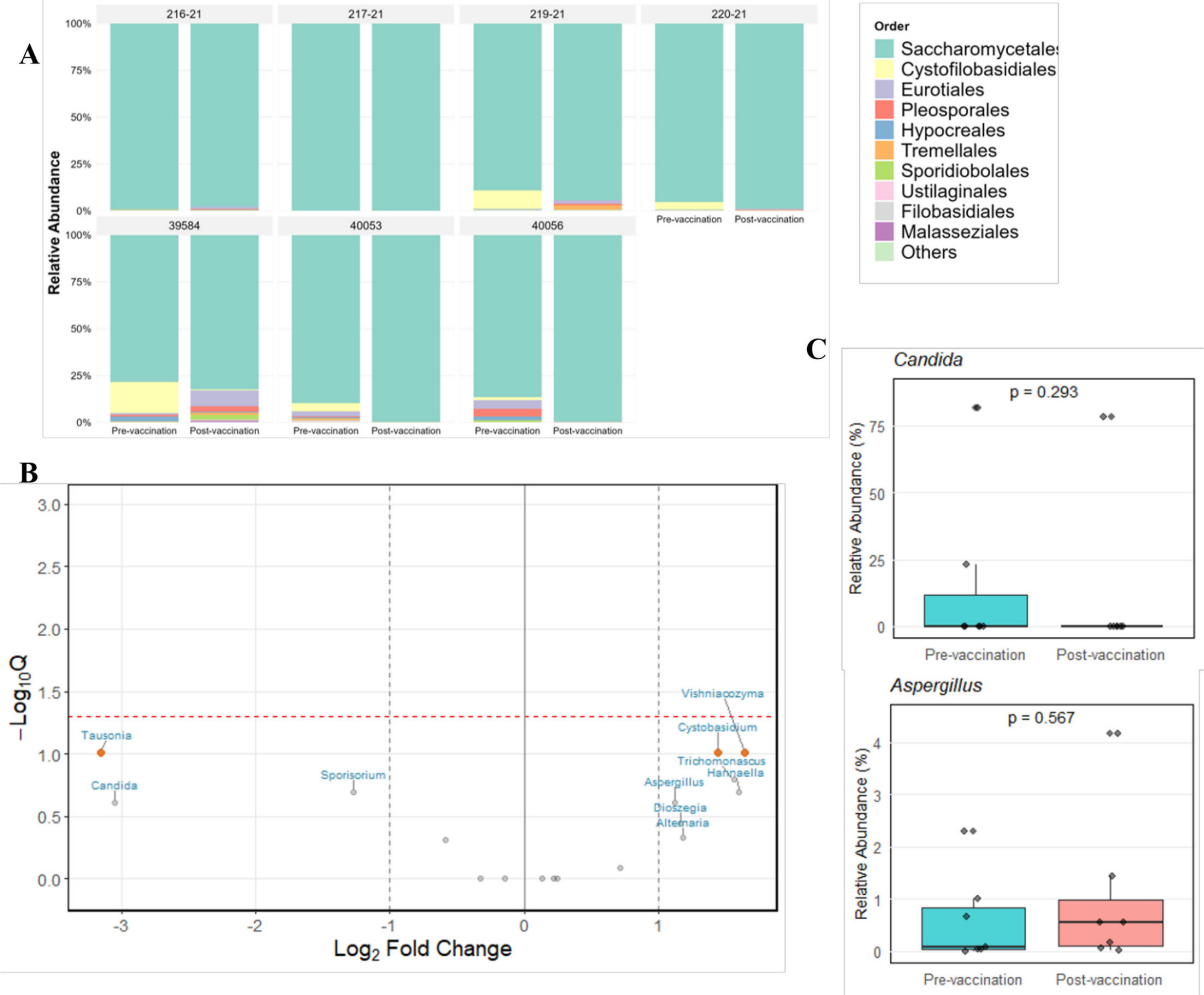
Taxonomic composition and differential abundance analysis of the gut mycobiome before and after vaccination. (**A**) Relative abundance of top 10 order taxa (depicted by color) in pre- and post-vaccination samples. (**B**) Differential abundance analysis of genera pre- and post-vaccination using ANCOM-BC2. The red dashed line indicates the false discovery rate threshold (*q* = 0.05). No genera showed significant differential abundance post-vaccination. Taxa with |Log_2_ Fold-change| > 1 are labeled in blue and the top three with the lowest *q* value are shown in orange. (**C**) Relative abundance of *Candida* (top panel) and *Aspergillus* (bottom panel) showed no significant changes between timepoints. Statistical comparisons were performed using Wilcoxon signed-rank test.

To assess whether NXT-2 vaccination resulted in changes at various taxa levels in the mycobiome, the relative abundance at the order level across timepoints was profiled. In the Japanese macaques, Saccharomycetales dominated the mycobiome in all animals at both timepoints, representing over 75% of the relative abundance in most samples with minor contributions from other taxa including Cystofilobasidiales, Eurotiales, and Pleosporales (Fig. 3A). The relative abundance profiles within animals appeared largely conserved with no consistent shifts observed between timepoints. The mycobiome of rhesus macaques showed greater order diversity, which was mostly dominated by Pleosporales (Fig. S2C). There were variable representations of other taxa including Saccharomycetales, Sporidiobolales, and Eurotiales. Despite these observed variations, the community structure within the individual animals remained stable following vaccination, with the exception of animal 38,480 which had a decrease in Saccharomycetales and an increase in Sporidiobolales and Trichosporonales post-vaccination.

Differential abundance analysis using ANCOM-BC2 showed that no genera was significantly enriched or depleted following NXT-2 vaccination in either the Japanese (Fig. 3B) or rhesus (Fig. S2D) macaque cohorts. In the Japanese macaques, fungal genera including *Tausonia*, *Candida*, *Sporosorium*, *Aspergillus*, *Cytobasidium,* and *Vishniacozyma* exhibited at least twofold change (|log₂ FC| > 1) (Fig. 3B). In the rhesus macaque cohort, genera including *Cutaneotrichosporon*, *Mucor*, *Candida*, *Fusarium,* and *Vishniacozyma* exhibited at least twofold change (|log₂ FC| > 1) (Fig. S2D). Notably, *Vishniacozyma* showed opposite directional fold change where the relative abundance was decreased in the Japanese macaques but increased in the rhesus macaque cohort. Given that NXT-2 has been shown to be protective against *Candida* and *Aspergillus* infections in mouse models, the relative abundance of these specific genera was assessed. The relative abundance of *Candida* and *Aspergillus* remained stable across vaccination timepoints in both the Japanese macaques (Fig. 3C, *Candida*: *P* = 0.293, *Aspergillus*: *P* = 0.567) and rhesus macaques (Fig. S2E, *Candida*: *P* = 1.000, *Aspergillus*: *P* = 0.281). These results demonstrate that NXT-2 vaccination does not significantly alter the taxonomic composition of the gut mycobiome.

### The mycobiome functional capacity is maintained following NXT-2 vaccination

**Fig 4:**
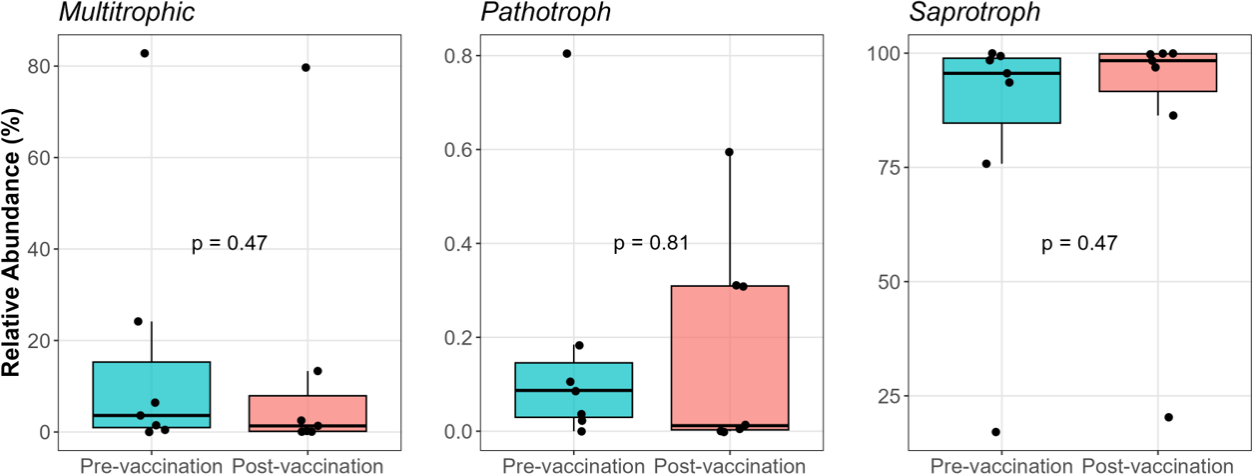
Functional guild composition of the gut mycobiome remained unaltered following NXT-2 immunization. Relative abundance of fungal trophic mode assigned using FUNGuild showing Multitroph, Pathotroph, and Saprotroph. There were no significant changes in functional guild abundance. Statistical comparisons between time points were performed using the Wilcoxon rank-sum test.

To assess whether the functional capacity is affected post-vaccination, FUNGuild was used to assign trophic modes (multitrophs, pathotrophs, and saprotrophs) to the fungal taxa. In Japanese macaques, saprotrophs dominated the mycobiome at both timepoints consisting of over 80% of the functional community (Fig. 4). Multitrophic fungi represented a minor component (about 5%), while pathotrophs were present in low abundance (<1%). Comparison of guild abundance between timepoints showed no significant changes in any functional category (Fig. 4; Multitrophic: *P* = 0.47, Pathotroph: *P* = 0.81, Saprotroph: *P* = 0.47). In rhesus macaques, multitrophic fungal taxa dominated the mycobiome with an average relative balance of 55% pre-vaccination and 87% post-vaccination. Saprotrophs represented a minor component, while pathotrophs were present in low abundance. Despite these differences in functional guild composition, NXT-2 vaccination did not significantly alter guild abundance in rhesus macaques between timepoints (Fig. S3; Multitrophic: *P* = 0.12, Pathotroph: *P* = 1.0, Saprotroph: *P* = 0.12). These results collectively show that NXT-2 vaccination did not alter the functional guild composition or ecological roles of the gut mycobiome.

### Changes in the gut microbiome diversity and composition following NXT-2 vaccination

**Fig 5:**
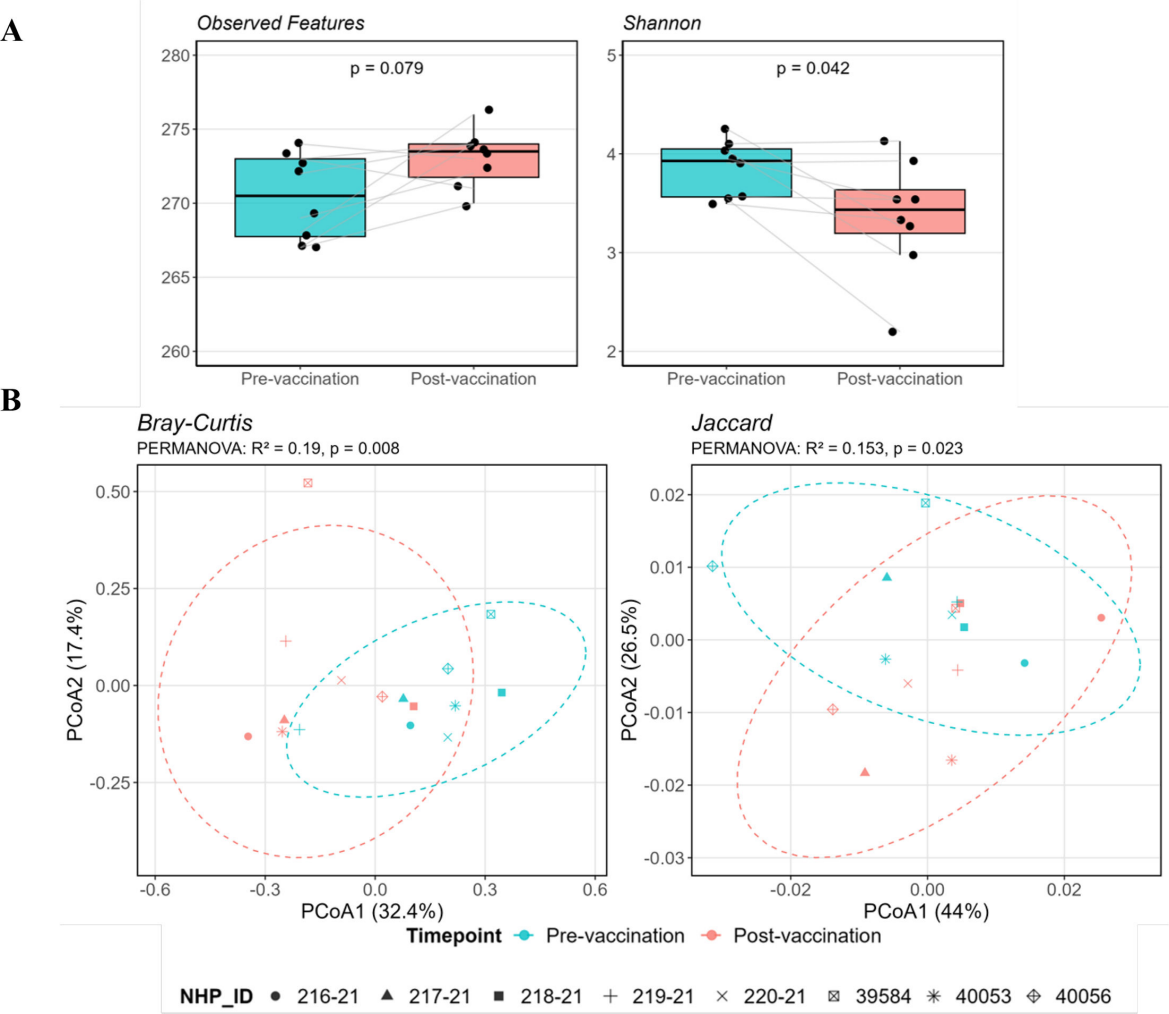
Alpha and beta diversity of the gut microbiome following NXT-2 vaccination. (**A**) Alpha diversity metrics (Observed Features and Shannon) before and after NXT-2 immunization in Japanese macaques (*n* = 8). Individual animals are shown as points connected by lines. Statistical comparison was performed using the Wilcoxon signed-rank test. (**B**) Beta diversity analysis (Bray–Curtis and Jaccard) visualized by PCoA showed statistically significant community shift. Individual samples are shown as shapes and colored by timepoint with 95% confidence ellipses. Group differences were assessed using PERMANOVA.

To assess whether NXT-2 vaccination altered the gut bacterial community and composition, alpha and beta diversity analyses were performed. In the Japanese macaques, the average observed features were 270 (range: 270–276) pre-vaccination and 273.5 (range: 270–276) post-vaccination although this change was not statistically significant (Fig. 5A, *P* = 0.079). For the Shannon index, there was a decrease from an average of 3.93 (range: 3.55–4.25) pre-vaccination to 3.44 (range: 2.20–4.13) post-vaccination (Fig. 5A, *P* = 0.042). Beta diversity analysis showed statistically significant differences in community composition between timepoints by both the Bray-Curtis (*P* = 0.008) and Jaccard (*P* = 0.0023) indices (Fig. 5B). However, PCoA plots for the beta diversity metrics showed substantial overlap between timepoint samples (Fig. 5B). In rhesus macaques, the bacterial community remained stable following vaccination. Observed features showed no significant changes from 400 (range: 208–408) pre-vaccination to 398 (range: 365–405) post-vaccination (Fig. S4A, *P* = 1.0), and Bray-Curtis also showed no significant differences in community composition (Fig. S4B, *P* = 0.660). Collectively, these results show that there are minimal changes to the gut microbiome with the majority of the community diversity being maintained across samples post-vaccination.

### Microbiome taxonomic composition remained stable with no differentially abundant taxa

**Fig 6:**
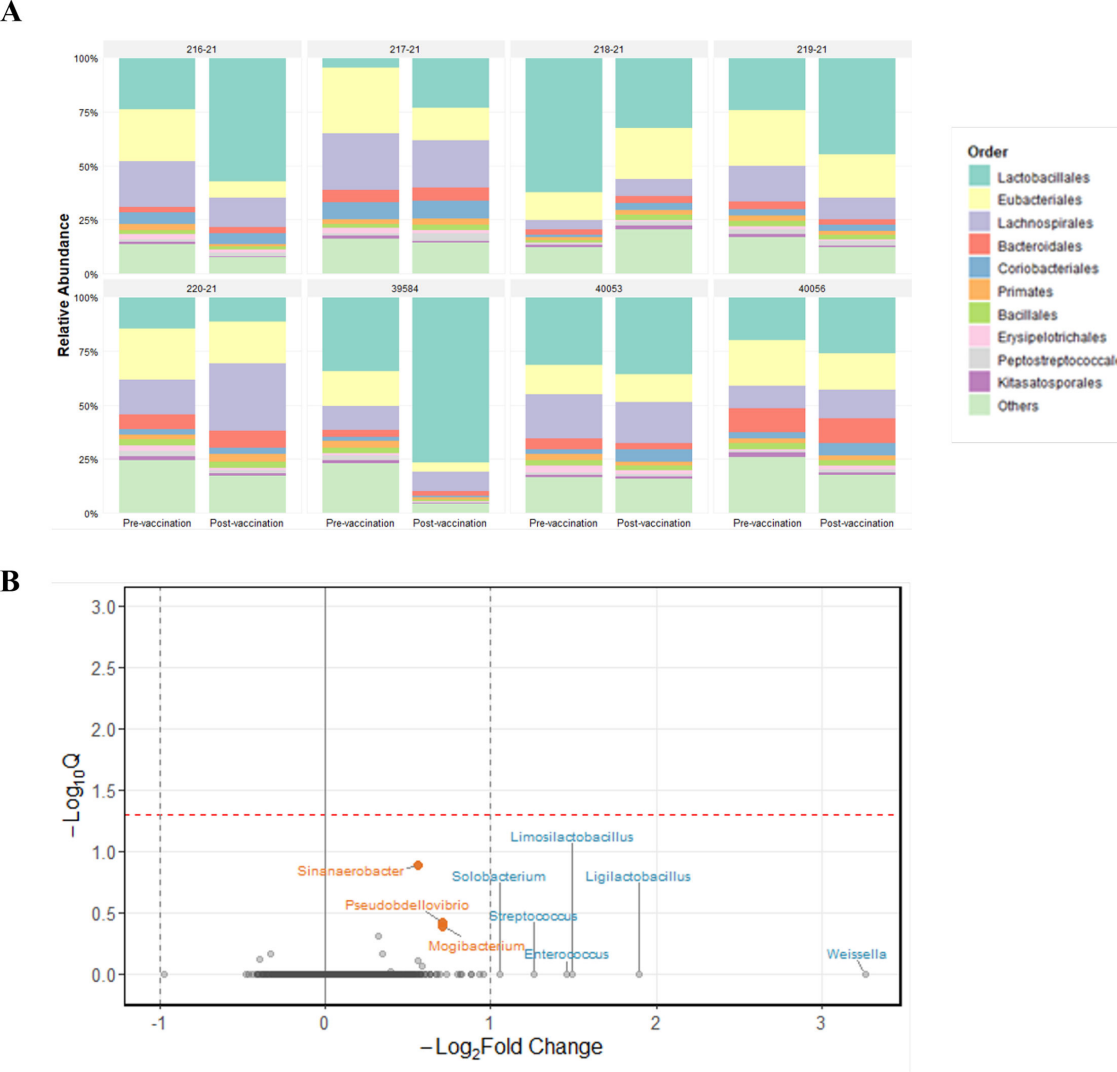
Taxonomic composition and differential abundance analysis of the gut microbiome before and after vaccination. (**A**) Relative abundance of top 10 order taxa (depicted by color) in pre- and post-vaccination samples. (**B**) Differential abundance analysis of genera pre- and post-vaccination using ANCOM-BC2. The red dashed line indicates the threshold (*q* = 0.05). No genera showed significant differential abundance post vaccination. Taxa with |Log_2_ Fold-change| > 1 are labeled in blue, and the top 3 with the lowest *q* value are shown in orange.

Given the modest but significant shifts in bacterial Shannon index and beta diversity metrics observed in the Japanese macaques, a taxonomic composition analysis and differential abundance testing were performed to identify which specific taxa were driving these changes. In Japanese macaques, taxonomic analysis at the order level revealed inter-animal variation in bacterial community composition, with dominant orders including Lactobacillales, Eubacteriales, Lachnospirales, and Bacteroidales (Fig. 6A). Individual animals exhibited diverse baseline compositions with varying proportions of Coriobacteriales, Erysipelotrichales, Peptostreptococcales, Bacillales, and other orders. Despite this individual variation, the relative representation of major orders remained largely consistent within individuals between pre- and post-vaccination timepoints. Rhesus macaques similarly displayed inter-individual variation in taxonomic composition, with dominant orders also including Lactobacillales, Bacteroidales, Eubacteriales, and Lachnospirales (Fig. S4C). As with Japanese macaques, individual rhesus macaques maintained relatively stable order-level composition between timepoints.

Differential abundance analysis using ANCOM-BC2 identified no significantly enriched or depleted genera following vaccination in either Japanese macaques (Fig. 6B) or rhesus macaques (Fig. S2D). In Japanese macaques, although several genera showed fold changes, including *Weissella*, *Ligilactobacillus*, *Limosilactobacillus*, *Enterococcus,* and *Streptococcus*, none reached statistical significance (all *q* > 0.05). The genera closest to reaching significance had less than twofold change. Similarly, in rhesus macaques, genera such as *Anaerobaculum* and *Catenibacterium* exhibited twofold changes but did not achieve significance (all *q* > 0.05). These results indicate that despite modest changes in overall community diversity observed in Japanese macaques (Fig. 5), NXT-2 vaccination does not significantly alter the abundance of individual bacterial taxa in either cohort.

### Functional capacity of the bacterial microbiome is maintained despite modest diversity shifts

**Fig 7:**
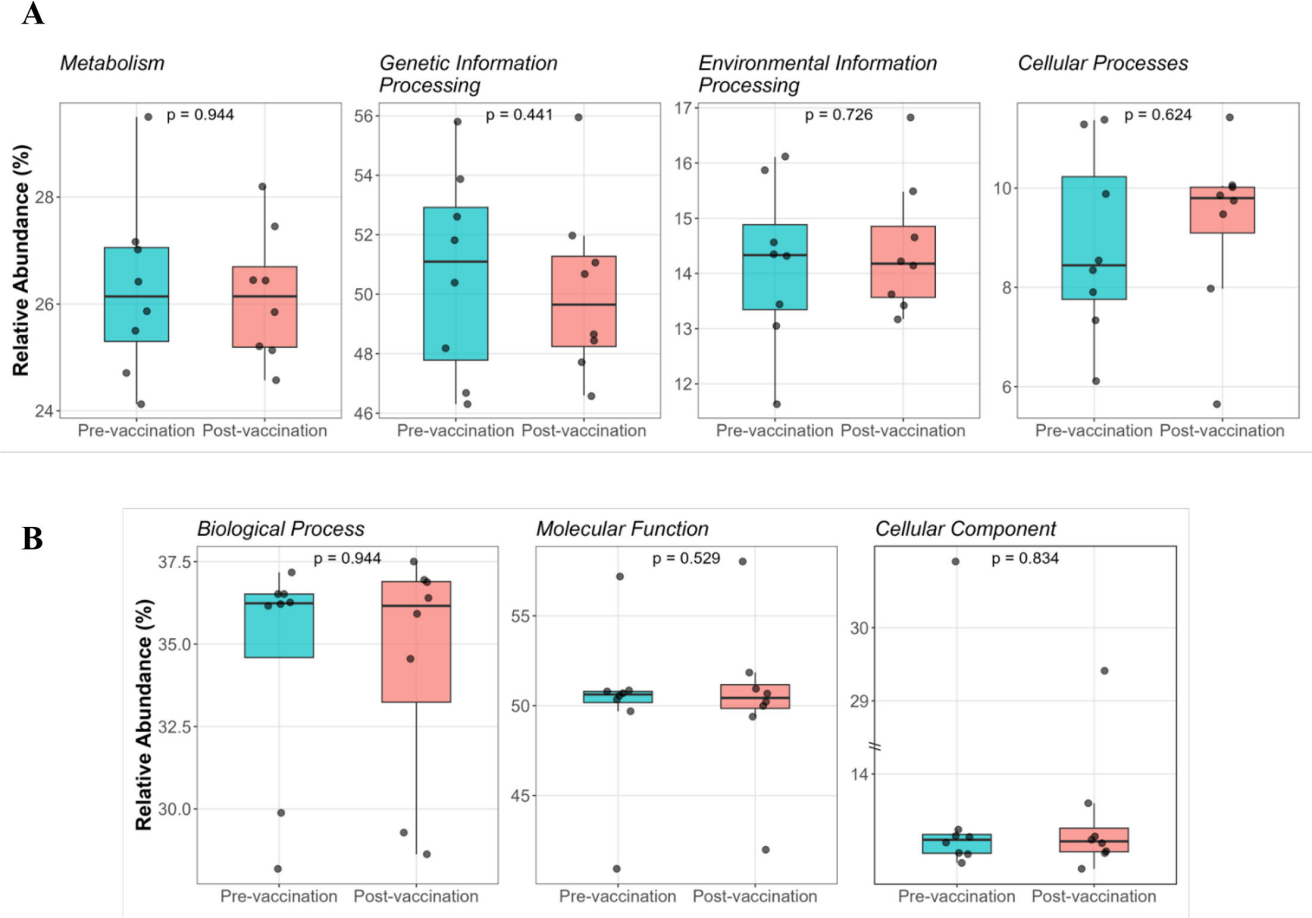
Functional pathway composition of the gut microbiome remains unchanged following NXT-2 immunization. Functional annotations using (**A**) KEGG and (**B**) Gene Ontology showing relative abundance at the major functional categories. There were no significant changes detected in any functional category pre- and post-immunization. Statistical comparisons between time points were performed using the Wilcoxon rank-sum test.

To assess whether the observed changes in bacterial diversity corresponded to alterations in functional capacity, we analyzed predicted functional pathways using KEGG and Gene Ontology classifications. In Japanese macaques, KEGG pathway analysis revealed no significant changes in any major functional category following vaccination, including metabolism (*P* = 0.944), genetic information processing (*P* = 0.441), environmental information processing (*P* = 0.726), and cellular processes (*P* = 0.624) (Fig. 7A). Similarly, Gene Ontology analysis showed stable relative abundances across all categories, including biological process (*P* = 0.94), molecular function (*P* = 0.53), and cellular component (*P* = 0.83) (Fig. 7B).

Rhesus macaques exhibited comparable functional stability, with no significant changes in KEGG pathways; metabolism (*P* = 0.584), genetic information processing (*P* = 0.584), environmental information processing (*P* = 0.100), and cellular processes (*P* = 0.100) (Fig. S4A) or Gene Ontology categories; biological process (*P* = 0.584), molecular function (*P* = 0.855), cellular component (*P* = 0.855) (Fig. S4B). These results demonstrate that despite modest shifts in community diversity metrics observed in Japanese macaques, the functional capacity of the gut bacterial microbiome remains stable following NXT-2 vaccination in both macaque cohorts.

## DISCUSSION

The increasing incidence of fungal infections has become a serious public health concern, prompting the need to develop novel therapeutics such as vaccines. To address this problem, our lab has developed the “pan-fungal” vaccine NXT-2 that has shown protective efficacy against multiple fungal infections (9, 12–14). In this study, we assessed the impact of NXT-2 vaccination on gut mycobiome and microbiome compositions and functions in independent cohorts of Japanese and rhesus macaques. To our knowledge, this is the first study to evaluate both fungal and bacterial communities in assessing a vaccine’s impact on the gut microbial ecosystem. We confirmed the immunogenicity of NXT-2 in these NHP cohorts and that fecal sample collected at post-vaccination timepoint was at peak antibody titer. The prime-boost interval was 4 weeks in the rhesus macaques and 6 weeks in the Japanese macaques, where a longer interval was explored as a strategy to enhance antibody magnitude and durability. Despite this difference, antibody responses were comparable between both cohorts. We show that despite inducing a robust systemic antibody response, NXT-2 vaccination maintains gut microbial homeostasis across both mycobiome and microbiome by preserving community structure across diversity, taxonomic composition, and functional capacity.

The gut mycobiome remained stable following NXT-2 immunization with no significant changes in alpha and beta diversities, taxonomic composition, or functional guild distributions in either Japanese or rhesus macaques. While several genera showed twofold changes, none were statistically significant. Notably, the bidirectional fold change of *Vishniacozyma* between macaques (decrease in Japanese, increase in rhesus) indicates that the observed fold changes possibly reflect individual or species-specific variation rather than vaccine-driven effects. This comprehensive stability is significant given that the mycobiome is less stable and more susceptible to environmental perturbations (36, 37). Studies performed in mice and humans have shown high variability in mycobiome communities between individuals and within the same individual overtime (36, 38). Although we observed variability in the mycobiome communities between individual animals at the order level, particularly in the rhesus macaques, the individual mycobiome profiles remained relatively stable post-vaccination. We observed that the mycobiome of both macaque cohorts was dominated by Ascomycota and Basidiomycota, which is consistent with studies performed in humans and other non-human primates (39–41).

The preservation of commensal fungi including *Candida* despite robust systemic and localized antibody responses (14) denotes that this vaccine can elicit protective immunity without disrupting fungal communities. This finding has important implications for vaccine efficacy, as *Candida* can act as either a beneficial commensal or opportunistic pathogen (42, 43). NXT-2 has shown protective efficacy against fungal infections in mucosal sites including the respiratory tract (9, 13) and vaginal tract (14) in preclinical animal models. This selective preservation of gut commensals despite mucosal immunity suggests that NXT-2 antibodies preferentially target pathogenic fungal morphotypes during invasive infections rather than commensal forms (44, 45).

Furthermore, the functional capacity of the mycobiome remained unchanged after NXT-2 vaccination. FUNGuild analysis revealed stable distributions of functional guilds were maintained following vaccination (46). These guilds collectively maintain diverse ecosystem functions including nutrient cycling, microbial community structure, and cross-kingdom interactions that support gut ecosystem homeostasis (19, 47). Commensal fungi provide ongoing immune training through exposure to fungal antigens that prime innate immunity (37, 48–50). The preservation of these functional guilds indicates that NXT-2 vaccination maintains both taxonomic composition and the functional capacity of the gut mycobiome, supporting comprehensive microbiome homeostasis.

The gut microbiome exhibited minimal changes in diversity, taxonomic composition, and functional capacity following NXT-2 vaccination. In Japanese macaques, although a significant decrease in the Shannon index and shifts in beta diversity were observed, the number of observed features remained unchanged. PCoA plots showed substantial overlap between timepoints, and no genera were significantly enriched or depleted following vaccination, indicating overall stability of the bacterial community despite fold changes in several taxa. Rhesus macaques showed complete stability in diversity metrics and differential abundance analysis. The dynamic nature of the gut ecosystem allows natural temporal variation in healthy individuals, where compositional shifts within individuals are expected but the majority of variation is observed between individuals (51–53). This suggests that the shifts we observed were primarily due to individual variations rather than a vaccine-driven effect. The observed taxonomic composition in both macaque cohorts is consistent with characterizations of healthy gut microbes in humans and other NHPs. The gut microbiome was dominated by organisms belonging to the Firmicutes (Bacillota) and Bacteroidetes (Bacteriodota) which is characteristic of a healthy gut of macaques where these two phyla represent over 80% of the total bacterial community (54–56). This phylum-level dominance is conserved among primates and can also be observed in humans where the Firmicutes and Bacteroidetes ratio has been used as a predictor of age and health status (57, 58).

The substantial variation of the microbiome observed in individual animals at the order level in this study is also indicative of a healthy gut where variation is observed between individuals, but the individual-specific microbial communities remain stable over time (51, 54). Beyond taxonomic stability, the functional capacity of the gut microbiome remained stable in both macaque cohorts after NXT-2 vaccination. Both KEGG and GO pathway analyses showed that there are no significant changes in major functional categories and capacities consistent with a healthy microbiome (59, 60). This functional stability indicates that essential microbiome processes critical for host health including immune regulation, maintenance of intestinal barrier function, and digestion are maintained (61–63). The preservation of both taxonomic composition and functional capacity demonstrates that the modest diversity shifts observed in Japanese macaques represent natural variation and not dysbiosis, as the bacterial community retained the diverse functional capacity essential for gut ecosystem homeostasis and host-microbiome interactions.

The growing burden of fungal infections together with the increase in populations at risk of serious fungal infections highlights a critical need to develop novel therapeutics (1, 4). The current antifungal drugs are limited and can cause treatment-limited toxicity, and there is increasing concern over the development of drug resistance (2, 3, 64). Apart from these challenges, current antifungal drugs have been shown to disrupt fungal and bacterial communities in the gut (19). Fluconazole administration in mice caused the restructuring of the mycobiome composition by depleting Ascomycota and expanding Mucormycota phyla. This resulted in the reduction of the microbiome diversity of the gut (65). In contrast to this antifungal-induced perturbations, NXT-2 vaccination has been shown to be protective against multiple fungal infections and without compromising the stability of the gut mycobiome and microbiome. Research on vaccine-microbiome interactions has largely concentrated on how pre-existing microbial composition influences vaccine efficacy; the question of whether vaccines disrupt gut ecosystems remains relatively unexplored. One study using shotgun metagenomic analyses demonstrated complete preservation of compositional, diversity, and functional stability across diverse patient populations with COVID-19 mRNA vaccine (24). NXT-2 demonstrates similar microbiome stability. Importantly, this represents the first comprehensive analysis of both bacterial and fungal communities following antifungal vaccination, demonstrating that protective immunity can be achieved without disrupting either microbial community.

While these findings are informative, there are limitations. The single pre-vaccination and post-vaccination timepoint sampling design provides a snapshot of endpoint stability during the period of peak immune response to the vaccine but does not capture potential long-term mycobiome and microbiome dynamics. Future studies would also address important variables, such as age, sex, and baseline microbiome profiles. Expanding microbiome assessment beyond the gut to include oral and vaginal ecosystems would evaluate systemic stability, particularly relevant for mucosal fungal communities and infections. Critically, evaluation in immunocompromised populations would provide important information on the effect of immunization on the microbiome in those most vulnerable to invasive fungal infections and therefore most likely to benefit from vaccination.

In conclusion, we demonstrate that NXT-2 vaccination elicits a robust antibody response that does not disrupt the gut mycobiome and microbiome in terms of diversity, taxonomic composition, and metabolic or functional capacity. Importantly, fungal organisms against which NXT-2 has demonstrated protective efficacy (including *Candida* and *Aspergillus*) (9, 12, 14) remained stable, indicating that protective immunity does not disturb resident fungal communities. This study provides the first comprehensive cross-kingdom analysis of the stability of gut microbial community in response to immunization with a fungal vaccine candidate. This in-depth analysis provides a template for evaluations of future therapeutics, especially interventions where off-target microbiome or mycobiome effects may be of concern.

## Data Availability

The data used and analyzed in the study will be available upon reasonable request to the corresponding author.

## SUPPLEMENTAL MATERIAL

Supplemental figures spectrum.01047-26-s0001.docx Figures S1 to S7.

ASM does not own the copyrights to Supplemental Material that may be linked to, or accessed through, an article. The authors have granted ASM a non-exclusive, world-wide license to publish the Supplemental Material files. Please contact the corresponding author directly for reuse.
