# Supplementary material for: Evaluating the impact of immunization with the “pan-fungal” vaccine, NXT-2, on the gut mycobiome and microbiome in non-human primates (NHPs)
Source: Microbiol Spectr. 2026 Jul 15;14(8):e01047-26. doi: 10.1128/spectrum.01047-26 (PMC13435867; doi:10.1128/spectrum.01047-26)
Supplement: Supplementary file 1 [file spectrum.01047-26-s0001.docx]

**C**

**B**

**A**

Figure S1: **NXT-2 immunization elicits robust humoral responses in Rhesus macaques**. **(A)** Study design showing timeline for NXT-2 immunization (1st and 2nd dose), fecal sample collection [Pre-vaccination (week -1) and Post-vaccination (week 6)], blood collection (red drops) and downstream analysis. NXT-2-specific plasma IgG responses measured as reciprocal endpoint titers (RET) over time; **(B)** mean titer ± SD **(C)** individual animal responses.

**C**

**B**

**A**

**E**

**D**

Figure S2: **The composition of the gut mycobiome in Rhesus macaques remained unaltered following NXT-2 vaccination**. **(A)** Alpha (Observed Features) and **(B)** Beta (Bray–Curtis) diversity metrics showed no significant changes following NXT-2 vaccination. **(C)** Relative abundance of top 10 order taxa (depicted by color) in pre- and post-vaccination samples. **(D)** Differential abundance analysis of genera pre- and post-vaccination using ANCOM-BC2. The red dashed line indicates the false discovery rate threshold (q = 0.05). No genera showed significant differential abundance post vaccination. Taxa with |Log_2_ Fold-change| >1 labelled in blue and top 3 with the lowest q value shown in orange. **(E)** Relative abundance of Candida (top panel) and Aspergillus (bottom panel) showed no significant changes between timepoints. Statistical comparisons were performed using Wilcoxon signed-rank test for **(A)** and **(E)**, and PERMANOVA was performed for **(B)**.

Figure S3: **Functional guild composition of the gut mycobiome of Rhesus macaques remained unaltered following NXT-2 immunization. (A)** Relative abundance of fungal trophic mode assigned using FUNGuild showing Multitroph, Pathotroph and Saprotroph. There were no significant changes in functional guild abundance. Statistical comparisons between time points were performed using the Wilcoxon rank-sum test.

**C**

**B**

**A**

**D**

Figure S4: **The composition of the gut microbiome of Rhesus macaques remained unaltered following NXT-2 vaccination**. **(A)** Alpha (Observed Features) and **(B)** Beta (Bray–Curtis) diversity metrics showed no significant changes following NXT-2 vaccination. **(C)** Relative abundance of top 10 order taxa (depicted by color) in pre- and post-vaccination samples. **(D)** Differential abundance analysis of genera pre- and post-vaccination using ANCOM-BC2. The red dashed line indicates the false discovery rate threshold (q = 0.05). No genera showed significant differential abundance post vaccination. Taxa with |Log_2_ Fold-change| >1 labelled in blue. Statistical comparisons were performed using Wilcoxon signed-rank test for **(A)** and PERMANOVA was performed for **(B)**.

**A**

**B**

Figure S5: **Functional pathway composition of the gut microbiome of Rhesus macaques remains unchanged following NXT-2 immunization.** Functional annotations using **(A)** KEGG and **(B)** Gene Ontology showing relative abundance at the major functional categories. There were no significant changes detected in any functional category pre and post immunization. Statistical comparisons between time points were performed using the Wilcoxon rank-sum test.

.
